# Monitoring changes in the landscape water balance: validation of satellite- and model-based evapotranspiration data in Lusatia, Germany

**DOI:** 10.1007/s10661-026-15120-8

**Published:** 2026-03-06

**Authors:** Jenny Kröcher, Gohar Ghazaryan, Uwe Spank, Beate Zimmermann, Frank Beyrich, Ottfried Dietrich, Christian Markwitz, Justus van Ramshorst, José Ángel Callejas-Rodelas, Gunnar Lischeid

**Affiliations:** 1 Leibniz Centre for Agricultural Landscape Research, Müncheberg, Germany; 2 Institute of Environmental Sciences and Geography, University of Potsdam, Potsdam, Germany; 3 Geography Department, Humboldt-University of Berlin, Berlin, Germany; 4 Chair of Hydrogeology and Hydrochemistry, Technische Universität Bergakademie Freiberg, Freiberg, Germany; 5 Institute of Hydrology and Meteorology, Dresden University of Technology, Dresden, Germany; 6 Research Institute of Post-Mining Landscapes, Finsterwalde, Germany; 7 German Meteorologcial Service (Deutscher Wetterdienst, DWD), Meteorological Observatory Lindenberg – Richard-Aßmann-Observatory (MOL-RAO), Lindenberg, Germany; 8 Bioclimatology, Faculty of Forest Sciences and Forest Ecology, University of Göttingen, Göttingen, Germany; 9 Quanterra Systems Ltd., Exeter, UK

**Keywords:** Evapotranspiration, Remote sensing, Eddy covariance, Lysimeter, Landsat, MODIS

## Abstract

Proper estimates of evapotranspiration rates and long-term totals of evapotranspiration (ET) are crucial for scientific research and practical issues such as sustainable water resources management and resilient land use planning. Traditionally, ground-based ET measurements and observations are used, which are precise and accurate but lack broad spatial coverage. Satellite-based ET products provide spatially comprehensive estimates, although the accuracy of products in certain regions and of certain land use types remains unclear. This study therefore presents a systematic multi-product validation of three publicly available satellite- and model-based ET products (CERv2, MODIS Terra Net Evapotranspiration (MOD16) and Landsat Provisional Actual Evapotranspiration Science Product), including two recently developed products that have not yet been comprehensively evaluated using long-term in situ measurements from lysimeters and eddy covariance sites in an anthropogenically shaped region such as the German lowland region. The lowest relative deviations from measured ET were found at grassland sites (Median relative deviation: 5–54%; Root Mean Square Difference (RMSD): 0.58–1.02 mm d^−1^). Across all three datasets, MOD16 showed the lowest deviations at nearly all sites (Median relative deviation: 10–54%; RMSD: 0.58–0.90 mm d^−1^). In addition to validating satellite- and model-based ET using in situ measurements, we evaluated whether the ET products can reliably represent the spatial and temporal dynamics of ET to assess their suitability for regional water management and hydrological applications. For this purpose, principal component analyses (PCA) of the time series of each satellite- and model-based dataset were performed. The results show comparable spatial and temporal patterns that can be attributed to land use, water availability and long-term land use changes. Yet, differences between products and land use types became evident in the absolute ET values, even though the PCA revealed consistent dominant patterns across datasets. This suggests that the characteristic spatial and temporal patterns are consistently and reliably represented by all three datasets, regardless of underlying modelling approaches and resolution. The findings highlight the practicability of satellite- and model-based ET estimates for analysing regional and mesoscale ET patterns while also revealing their limitations in estimating absolute values due to model assumptions and spatial aggregation effects.

## Introduction

Evapotranspiration (ET) is central to ecosystem functioning by regulating the exchange of water and energy between the land surface and the atmosphere. By controlling moisture availability and influencing local microclimates, ET is linked to photosynthetic activity and plays an important role in plant growth (Fisher et al., 2017). Additionally, ET modulates surface temperatures and affects cloud formation (Miralles et al., 2014). As such, reliable ET measurements are crucial for assessing water resources, optimizing agricultural practices, and understanding broader climatic feedbacks (Fisher et al., 2017).

Despite its key role, quantifying ET and capturing its spatio-temporal variability remains one of the greatest challenges (Dembélé et al., 2020; Morton, 1983; Zhao et al., 2013). Ground-based methods such as eddy covariance (EC) and lysimeters are often resource and labour intensive, and they usually yield point- or field-scale observations that cannot adequately represent spatial heterogeneity (Oren et al., 2006). More recently, scintillometers have been demonstrated the potential to derive ET estimates based on optical and microwave scintillation measurements over path lengths of a few kilometres (e.g., Meijninger et al., 2002, 2006; Ward et al., 2015). Although this is considered a major progress, the spatial coverage of scintillometers is still far from the spatial coverage required to assess ET at the landscape or regional level.

Remote sensing and particularly satellite-based datasets have become an increasingly important tool for deriving ET estimates at local and global scales to overcome the spatial coverage limitations of in situ methods (Anderson et al., 2024; García-Santos et al., 2022; Kalma et al., 2008; Nouri et al., 2015; Zhang et al., 2016). A variety of satellite-based ET products have emerged, each employing different modelling approaches and input data (Zhang et al., 2016). For instance, physically based approaches, such as the widely applied MOD16 product based on the MODIS (Moderate Resolution Imaging Spectroradiometer) satellite sensor, combine satellite-derived vegetation indices with a Penman–Monteith framework to derive global ET estimates (Mu et al., 2011). Energy-balance-based approaches, such as the Atmosphere–Land Exchange Inverse product (ALEXI), the Mapping Evapotranspiration at High Resolution with Internalised Calibration (METRIC) and the Surface Energy Balance Algorithm for Land (SEBAL), use surface temperature retrievals from the thermal infrared range to separate sensible and latent heat fluxes (Abbasi et al., 2023; Allen et al., 2007; Anderson et al., 2012). Other datasets, such as the Global Land Evaporation Amsterdam Model (GLEAM) (Miralles et al., 2011), integrate microwave observations and soil moisture constraints to refine ET estimates, making them particularly suited for capturing variability across different vegetation types and climatic zones. In addition, reanalysis-driven products, like CERv2 for Central Europe, or global meteorological reanalysis ET data, such as ERA5 or MERRA-2, merge remote sensing observations with physically based atmospheric models to generate continuous time series at broader spatial scales (Hersbach et al., 2020; Somogyvári et al., 2024).

Collectively, these satellite-based datasets represent a trade-off between spatial and temporal resolution. High spatial resolution is particularly important for spatially distinguishing between different types of vegetation with characteristic seasonal evaporation dynamics or for capturing contrasts between irrigated fields, wetlands and dry sites. In addition, local water management measures such as groundwater extraction or small-scale renaturation measures often take place on fine spatial scales and over short periods of time, whereby reliable high-resolution ET data are a supporting element for evaluating the effectiveness of the implemented measures.

The MOD16 product provides 8-day global ET estimates at medium spatial resolution (500 m) and is thus suitable for large-scale watershed comparisons and for capturing seasonal and interannual variability in ET (Mu et al., 2013). To provide ET products for small-scale analyses, initiatives like the Water Productivity portal (WaPOR), developed by the Food and Agriculture Organization of the United Nations (FAO) (Blatchford et al., 2020) provide high-resolution ET data. Those datasets are primarily available for Africa and the Near East, supporting agricultural productivity and water resources management in areas with significant water scarcity, but are limited in their geographical coverage and global transferability. In contrast, Landsat-based ET data offer a unique advantage by providing global coverage at a 30-m resolution (Senay et al., 2023). However, these data are limited in their temporal resolution to a 16-day revisit cycle. Reanalysis derived datasets, in turn, offer high temporal resolution but have coarse spatial resolution (Hersbach et al., 2020). Further efforts have been made for the derivation of high resolution ET products based on downscaling of other datasets such as Sentinel 2 and 3 (Guzinski & Nieto, 2019).

Spatial and temporal resolution are not the only aspects to consider for selection of data for a particular analysis. Despite advancements, remote sensing-based ET estimation is inherently subject to uncertainties arising from factors such as atmospheric interference, cloud cover, and variations in land surface characteristics (FAO, 2023). Differences in spatial resolution, model assumptions and input data can lead to significant deviations of satellite-based ET estimates, which limit their applicability across different climatic zones and landscape types, necessitating calibration and validation to reflect local conditions accurately (Nouri et al., 2015; Sharma et al., 2016). Validation through in situ measurements, including applications like eddy covariance systems or lysimeters, is crucial for ensuring the accuracy of estimates, particularly for regional water management. Some effort towards comparison and validation has been done (Bai, 2023; Salazar-Martínez et al., 2022; Tran et al., 2023) but more is needed, especially including validation of datasets at multiple scales (García-Santos et al., 2022; Kite & Droogers, 2000; Xie et al., 2024) and in regions of intensive anthropogenic land use like the lowlands in Central Europe.

Previous studies have addressed related aspects but with important limitations. Casper et al. (2023) found that integrating satellite-based datasets improved the performance of hydrological models for two Central European catchments. However, their study did not include in situ validation, so potential systematic biases in satellite-based ET estimates under different land use were not addressed. Dąbrowska-Zielińska et al. (2024) compared various ET products, including ECOSTRESS, MODIS and ERA-5, for grassland areas in Poland. However, the comparison and assessment of the products was carried out indirectly by correlating them with soil moisture time series and vegetation height, rather than using in situ ET measurements. Fluhrer et al. (2025) compared various large-scale ET datasets with in situ measurements in Central Europe. However, they found that inaccuracies occur in spatiotemporally varying landscapes, that are not sufficiently resolved by the large-scale datasets.

In this context, systematic intercomparison and validation of fine-scale satellite- and model-based ET datasets, including recently developed regional reanalysis products and high-resolution optical sensors, remain largely lacking. In particular, the regional WRF-based CERv2 product and the Landsat-based ET dataset have not yet been comprehensively evaluated against long-term in situ measurements across contrasting land use types, despite their increasing relevance for applied hydrological studies due to their free availability and operational processing. The aim of this study is to compare three publicly available satellite- and model-based ET datasets (MODIS, Landsat and CERv2) with each other and with in situ measurements. The region of Lower Lusatia serves as a particularly relevant test case due to extensive landscape changes in recent decades including the flooding of open-cast mines, renaturation and reforestation, and the removal of vegetation in active open-cast mines, which strongly affect ET dynamics.

Beyond product-wise accuracy assessment, this study applies a principal component analysis (PCA) to investigate whether the datasets consistently reproduce the dominant spatio-temporal patterns of ET and their modification by land use, water availability and long-term landscape change. By combining site-scale validation with a multi-product, multi-scale and pattern-oriented analysis, this work provides new insights into the strengths and limitations of widely used ET datasets for monitoring landscape water balance dynamics in heterogeneous and human-impacted regions. Specifically, the following research questions will be addressed:

- How accurately do satellite- and model-based datasets capture ET and its spatio-temporal patterns under different land uses?
- To what extent do the satellite- and model-based data distinguish between the main effects of the spatio-temporal variability of evapotranspiration?

This study thus provides a sound basis for using such ET datasets for hydrological applications, land use planning and water management.

## Materials

### Study region

**Fig. 1:**
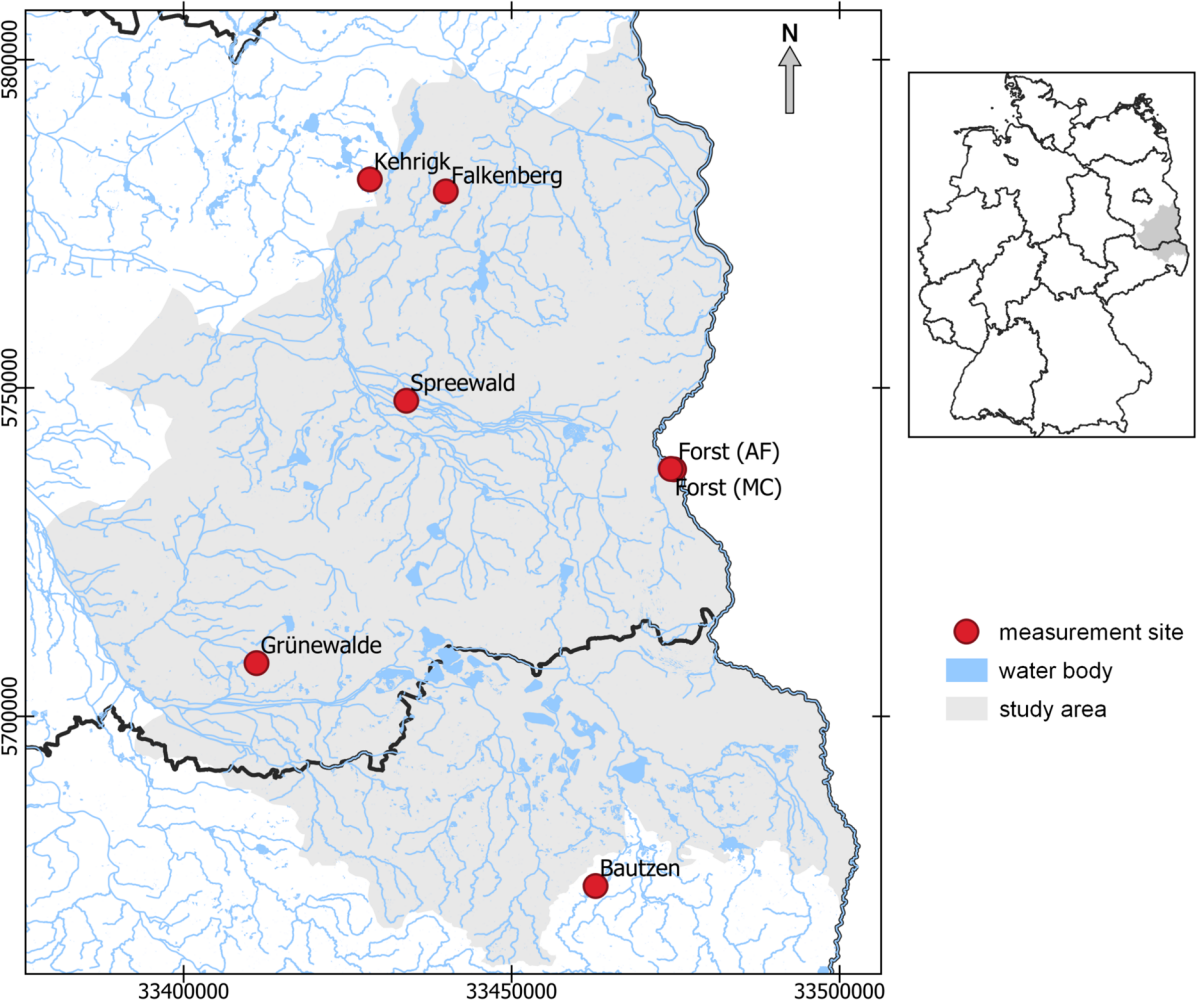
Study area and position of in situ ET measurement sites. Coordinates are given in UTM (EPSG: 25833, units: meters) to minimize distortions and ensure an accurate spatial representation of the study area

The study region is part of the Lower Lusatia Central European low-land area in Germany. It is located approximately 80 km south-east of Berlin and covers around 10,000 km^2^ (Fig. 1). The landscape is characterised by a rural settlement structure and a pattern of agricultural and forestry areas with embedded natural and artificial lakes and ponds. The landscape was shaped by Pleistocene glaciation. Glacial valleys developed with shallow groundwater levels, often resulting in the formation of large fens (now largely drained), as well as of outwash plains and moraines (Gerwin et al., 2023). These hydrological and geological features play a major role in regional ET dynamics by affecting soil water availability and vegetation patterns during the growing season, especially in dry years.

The climate can be classified as suboceanic with minor continental influence (Gädeke et al., 2017). The mean annual temperature and precipitation are 10.0 °C and 566 mm, respectively (1991–2020; weather station Cottbus (Deutscher Wetterdienst, 2023a, 2023b)). The region experiences a negative climatic water balance of approximately − 136 mm yr^−1^ (1991–2020; (Deutscher Wetterdienst, 2024), indicating that the theoretical water demand through potential evapotranspiration exceeds the precipitation input. The predominance of sandy soils with a small water storage capacity makes the region particularly susceptible to drought stress, which directly impacts ET rates (Krümmelbein et al., 2012; Reyer et al., 2012). In addition, the past decade has been characterized by a series of exceptionally dry years (e.g., 2018, 2019, 2020 and 2022), which have accentuated the water deficit in the region.

Based on CORINE Land Cover/Land use data, land use is dominated by forestry (44%), arable land (28%) and grassland (13%) (European Environment Agency, 2019a). Due to the sandy, low-yield soils and frequent water stress, Scots pine (*Pinus sylvestris*) is the predominant tree species (Hofmann & Pommer, 2005). Grassland is primarily found in glacial valleys on former peatlands. In addition, lignite open-cast mining has been shaping Lusatia for over 120 years, expanding significantly after the Second World War (Gerwin et al., 2023). It has left a 906 km^2^ footprint (Statistik der Kohlewirtschaft, 2024), whereas the corresponding lowering of the groundwater level has impacted the water balance at 3200 km^2^ (Uhlmann et al., 2020). Abandoned coal mines now constitute an extensive post-mining landscape with the largest artificial lake landscape in Europe (Gerwin et al., 2023; Hangen-Brodersen et al., 2005).

### Satellite- and model-based evapotranspiration datasets

Three satellite- and model-based ET data products (‘CERv2’, ‘MODIS’ and ‘Landsat’) differing in spatial and temporal resolution as well as with respect to methodology were tested (Table 1). Despite the differences, the three datasets each have advantages for specific hydrological objectives. The CERv2 dataset provides consistent coverage and high temporal resolution, while MODIS offers a well-established dataset with long time series, which is particularly valuable for analysing interannual variability and long-term hydrological trends. Landsat-based ET estimates have the advantage of high spatial resolution, which allows for detailed assessments of land use effects and small-scale land use changes. Together, the three products represent complementary strengths for hydrological applications, ranging from regional monitoring to local water management.

**Table 1:** Comparison of satellite- and model-based datasets with ET estimates used in this study

Dataset	Spatial resolution	Temporal coverage and resolution	Key features	References
CERv2	2 km	1980–2023 hourly	Generated by dynamic downscaling of meteorological and reanalysis data incorporating land cover, vegetation and soil moisture for ET estimation for the region of Berlin-Brandenburg	Jänicke et al., 2017; Bart et al., 2024
MODIS Evapotranspiration/Latent Heat Flux product	500 m	2001–2024 8 days	Penman–Monteith framework; incorporates MODIS LAI/FPAR, albedo, and land cover with reanalysis meteorological inputs (e.g., air temperature, radiation); designed for vegetated surfaces; validated with flux tower and watershed data	Mu et al., 2013; Running et al., 2021
Landsat Actual Evapotranspiration (ETa) Science Product	30 m	1982–2024 16 days	High-resolution ET from the SSEBOp model; combines land surface temperature, vegetation indices (NDVI), and auxiliary meteorological data; validated against flux tower data; provisional product with global availability	Senay, 2018; Senay et al., 2023

#### CERv2

The CERv2 dataset is based on the method and data used in Jänicke et al. (2017). It was specifically developed for the German federal states of Berlin and Brandenburg and generated by dynamic spatial downscaling with the Weather Research and Forecasting Model (WRF), version 4.3.3, and the ERA5 reanalysis data (Hersbach et al., 2020) provided by ECMWF (Bart et al., 2024). Unlike MODIS and Landsat, CERv2 is therefore not based on an energy balance or biophysical model, but on a physically based atmospheric model (WRF) that simulates ET indirectly from the coupled energy and water balance processes. The algorithm combines atmospheric drivers (derived from ERA5) with land cover, vegetation and soil water balance (Somogyvári et al., 2024).

The gridded dataset has been developed with a resolution of 2 km for the German federal states of Berlin and Brandenburg and a temporal resolution up to hourly values (Bart et al., 2024). ET is one of the hydro-meteorological variables calculated in this dataset for the period 1980–2023 (Somogyvári et al., 2024). So far, the dataset has been comprehensively validated with regard to near-surface air temperature and precipitation (Bart et al., 2024; Jänicke et al., 2017). The more than 30-year-long time series and comprehensive coverage of the area enclosed by the federal states of Berlin and Brandenburg make the dataset worth for considering as input data for hydrometeorological investigations (Somogyvári et al., 2024).

#### MODIS evapotranspiration/latent heat flux product

This study used the ‘MOD16AGF Version 6.1 Evapotranspiration/Latent Heat Flux’ product, a gap-filled 8-day composite dataset with a 500-m spatial resolution (Mu et al., 2013; Running et al., 2021). The core principle for calculating ET is the biophysical energy balance model based on Penman–Monteith (Mu et al., 2013). ET is calculated as the sum of transpiration, soil evaporation and evaporation from the canopy, controlled by (1) energy availability, (2) aerodynamic resistance and (3) surface resistance, which represents vegetation physiology and soil moisture. The algorithm uses MODIS remote sensing products (LAI, FPAR, albedo, land use) combined with meteorological reanalyses (air temperature, vapour pressure deficit, solar radiation) from NASA’s Global Modelling and Assimilation Office (GMAO) (Zhu et al., 2022).

The products’ ET values represent the sum of all eight days within the composite period. Based on the quality flag (ET_QC), we used pixels with good quality and discarded pixels that used a back-up algorithm or contained filled values for comparison with the in situ measurements. In addition, pixels with clouds were masked out.

#### Landsat

The Landsat Collection 2 Provisional Actual Evapotranspiration Science Product (Senay, 2018; Senay et al., 2023) provides a high spatial resolution of 30 m.

The core principle is a simplified energy balance model (SSEBop) that uses land surface temperature as a proxy for ET (Senay et al., 2022). The land surface temperature is based on thermal infrared data from Landsat and the Advanced Spaceborne Thermal Emission and Reflection Radiometer Global Emissivity Dataset (ASTER GED) (Petrakis et al., 2024). The SSEBop model also incorporates other parameters such as the normalised difference vegetation index (NDVI), air temperature, a digital elevation model (DEM), net radiation and reference evapotranspiration (Petrakis et al., 2024). The algorithm compares each pixel with a “hot” (dry) and a “cold” (wet) reference pixel and scales the ET from this (Nouri et al., 2015; Senay et al., 2022). The advantage is the high spatial resolution, while the disadvantage is that precipitation or air humidity are not directly taken into account (Senay et al., 2022).

The data product includes multiple outputs, such as daily ET (in mm), ET fractions, and uncertainty estimates, along with quality assessment layers. The uncertainty estimate is derived from the surface temperature uncertainty, which is based on the radiometric saturation and the pixel quality assessment.

For our analysis, we used the daily ET values and quantitative uncertainty estimates. As part of the internal processing conducted by United States Geological Survey, pixels affected by clouds, shadows, radiometric saturation or missing ASTER GED auxiliary data were masked. To ensure data quality, we only included satellite images with less than 50% cloud coverage. While preliminary validations show reasonable agreement with in situ measurements, such as those from EC measurement sites, the data remain provisional and are subject to further refinement (USGS, 2025).

### Land cover/land use datasets

The substantial change of land use in the study region during the last three decades is assumed to have had a major effect on ET. To account for that, area-wide information was derived from CORINE Land Cover/Land use data 2018 with 100 m spatial resolution (European Environment Agency, 2019a) and aggregated to eight land use classes (residential, open-cast mining, arable land, grassland, forest, peatlands and heathland, non-vegetated, water bodies). Data on land use change were derived from CORINE Land Cover/Land Use data datasets (European Environment Agency, 2019b, 2019c, 2019d, 2019e) between 1990 and 2018 and aggregated to 16 land use change classes based on aggregated land use classes.

### In situ evapotranspiration measurement data

In the study region, various in situ datasets on ET are available, which were collected over four to 16 years using lysimeters or eddy covariance (EC) stations. A total of seven datasets (Fig. 1) were included in the comparative analysis with the satellite- and model-based datasets (Table 2). The measurement sites are distributed on different types of land use and provide, therefore, comprehensive insights in relation to land use. Additionally, we collected soil moisture time series from site Kehrigk, Falkenberg and Lippen in the south of the study area, which were used for additional comparison with the satellite- and model-based data.

**Table 2:** Description of measurement site locations and data sampling methods

Name of dataset	Coordinates of data sampling (WGS84)	Land cover	Sampling method	Measurement period (used data)	Temporal resolution of measurements and data processing
Kehrigk	52.182°; 13.953°	Pine forest	Eddy-Covariance	2003–2014, 2018–2020	30 min fluxes based on eddy-covariance measurements with a USA-1 sonic anemometer and a LI7500 infrared gas analyser at 10 Hz sampling rate, processed as described in Beyrich and Adam (2007) and in Becker et al. (2024)
Falkenberg	52.166°; 14.124°	Grassland	Eddy-Covariance	2002–2016, 2018–2020	
Spreewald	51.879°; 14.040°	Grassland (Wetland)	Weighable lysimeter	2010–2022	10 min measurements which were aggregated to hourly and daily resolution to calculate ET as the residue of the water balance as described in Dietrich et al. (2016) and Dietrich (2024a, 2024b)
Forst (AF)	51.783°; 14.633°	Agroforestry (Cropland)	Eddy-Covariance	2016–2022	30 min measurements using lower-cost eddy covariance set-ups and data processing as described in Markwitz et al., (2020a, 2020b) for the years 2016 and 2017, and in Callejas-Rodelas et al. (2024) and van Ramshorst et al. (2024) for the years 2019 to 2024
Forst (AB)	51.783°; 14.633°	Arable land	Eddy-Covariance	2016–2022	
Grünewalde	51.517°; 13.719°	Grassland	Weighable lysimeter	2012–2022	Hourly measurements from 2012 to September 2021 and 5 min from September 2021 which were aggregated to hourly resolution to calculate ET as the residue of the water balance according to Dietrich et al. (2016)
Bautzen	51.217°; 14.468° (position of platform slightly changes over the years)	Reservoir	Eddy covariance	2018–2019, 2021–2022	30 min records of ET using a floating EC measurement station; Daily data were derived by temporal aggregation; Data failures were closed using the “REddyProcWeb online tool” (Wutzler et al., 2018). Only days with total gaps < 4 h and continuous gaps < 2 h were included. For details, see Spank et al., (2023, 2025)

## Methods

### Processing of satellite data

We extracted evapotranspiration values from the pixels of the three satellite- and model-based raster datasets and transformed them into time series for each pixel. The CERv2 and Landsat datasets were processed with the software R version 4.2.3 (R Core Team, 2023). We used the package ncdf4 (Pierce, 2024) to load the NetCDF files. Raster-to-table conversion was done using the packages terra (Hijmans, 2023) and sf (Pebesma, 2018; Pebesma & Bivand, 2023), and data wrangling with dplyr (Wickham et al., 2023). The MODIS dataset was accessed via Google Earth Engine (GEE) (Gorelick et al., 2017). The MODIS raster was converted directly into a pixel-wise time series table in GEE and then downloaded.

To account for different temporal resolution of the various products, we aligned the data to weekly time series in R. This aggregation was carried out not only for reasons of practical harmonisation, but also for hydrological considerations. Important processes that influence the long-term dynamics of evapotranspiration, such as changes in soil moisture and vegetation phenology, exhibit dynamics on a multi-day time scale. Weekly averaging also reduces the influence of short-term noise, e.g. due to cloud-related data gaps and daily variability in atmospheric demand. Aggregation to weekly values therefore offers a robust compromise between temporal resolution and signal stability across all ET products and provides the dominant seasonal and interannual ET dynamics relevant for water balance studies and hydrological applications. We aggregated the CERv2 data from hourly ET into weekly averages. If the MODIS and Landsat datasets provided one or more observations per week, we averaged daily to weekly values. Missing values were filled by linear interpolation. Time series with gaps longer than six months were excluded from further analysis.

We quantified the uncertainty associated with spatial resolution differences, we upscaled the 30-year averaged Landsat 30 m ET product to 500 m and 2 km and the 30-year averaged MODIS ET product to 2 km using area-weighted averaging of valid subpixels. We then computed the scale effect by calculating the mean difference and standard deviation of aggregated Landsat and MODIS grids with original 30 m Landsat and 500 m MODIS grids, respectively. Metrics were reported both for the whole study area and stratified by land-use classes.

### Processing of in situ data

The in situ data were collected using different measurement systems and were provided at different temporal resolutions (from 5 min to hourly). Therefore, each dataset was processed and aligned individually. Using eddy covariance data from Falkenberg, Kehrigk, Bautzen and Forst, actual ET was calculated from latent heat flux (LE) and latent heat of vaporization (λ) as a function of air temperature (T_air_):

1 $$ET=LE/\lambda$$

2 $$\lambda =\left(2.501-0.00237\cdot {T}_{air}\right)\cdot {10}^{6} J {kg}^{-1}$$

The processing of the lysimeter data included plausibility checks, the removal of invalid data and aggregation of 5-min and 10-min data to hourly values. The lysimeter sites at Spreewald and Grünewalde consist of four weighable lysimeters, each. For the comparative analysis we chose the time series of the lysimeters that represented the surrounding of the sites best to account for representativeness of the bigger footprint of the satellite data. For the site Spreewald, we chose the lysimeter that represented local groundwater dynamics. For the site Grünewalde, we chose the lysimeter that represented grassland cover and arenic regosol as soil type. ET was calculated as the residual of the water balance, expressed as:

3 $$ET=P-SW-\Delta S,$$

where P is the precipitation [mm h^−1^], SW is seepage water volume [mm h^−1^], and ΔS [mm h^−1^] represents the change in lysimeter weight, that is, the change in soil water storage over a given period. Precipitation was measured from positive weight differences for the lysimeters at the sites Grünewalde and Spreewald, respectively, assuming no ET occurs during rainfall as described in Dietrich et al. (2016). ET was calculated with hourly resolution and then summed to daily ET rates, if less than three hourly ET rates were missing.

All in situ datasets were aggregated to weekly mean values of daily ET, analogously to the aggregation of the satellite- and model-based datasets. Time series gaps were not gap-filled due to the risk of generating artefacts.

### Comparison of satellite- and model-based with in situ ET data

To compare the satellite- and model-based data with the in situ data, the raster pixel times series containing the measurement stations were selected automatically. Subsequently, this selection was visually checked to minimise influences from heterogeneity of land uses or soil types within the respective pixels, especially for the 2-km resolution CERv2 data. This representativeness check was based on the proportion of dominant land-use classes within each pixel. Land-use composition was derived from the regional land-use map, and a pixel was considered representative if the dominant land-use class within the pixel corresponded to the land use at the measurement site. If the automatically selected CERv2 pixel contained a substantial proportion of contrasting land-use types (e.g. forest–agriculture or land–water mixtures), neighbouring pixels within a 2-km radius were evaluated. A neighbouring pixel was selected only if it exhibited a clearly higher share of the target land-use class and more homogeneous surface characteristics. No further optimisation or averaging across multiple pixels was applied. This refinement affected only one site (Falkenberg). For all other sites, the automatically selected pixel was retained.

The satellite- and model-based datasets were validated with in situ measurements by comparing the ET values at the respective weekly dates and calculating the absolute differences. The deviation measures BIAS, Mean Absolute Deviation (MAD), Root Mean Squared Deviation (RMSD), Median Relative Deviation and the coefficient of determination (R^2^) were recorded as quality criteria.

### Principal component analysis for comparison of satellite- and model-based ET data

The availability of long-term satellite-based ET data enables to resolve spatial and temporal ET patterns due to different drivers like land use, meteorological conditions, soil properties and human effects. These patterns provide valuable information about regional hydrological dynamics and vegetation development. Beyond assessing absolute ET values, it is crucial to evaluate whether the datasets consistently capture relative spatial and temporal variations. Such patterns are particularly relevant for applications where relative differences rather than absolute ET values are required, for example in drought monitoring or in assessing the effects of land use changes on water balance. For an efficient comparison of the consistency and of the characteristics of these patterns across the three satellite- and model-based datasets, we applied a principal component analysis (PCA) of the ET time series for each satellite- and model-based dataset. PCA is a multivariate statistical method used to reduce complex datasets to a few independent (orthogonal) principal components (PC). These PCs summarise the common variance of the input data and make it possible to visualise dominant patterns in temporal and spatial structures. By reducing the data to a few principal components, it is possible to identify the key influencing factors and recurring dynamics without having to consider the entire data complexity.

The PCA requires synchronous, gapless time series. As a compromise between the different temporal resolutions of the satellite datasets, a weekly resolution was chosen for each dataset. By applying PCA to each satellite- and model-based dataset, the PCs of each analysis were derived in such a way that each subsequent, orthogonal PC explains the greatest possible variance of the dataset. Each PC thus represents a specific pattern or the effect that can be attributed to certain (physical) processes in the observed area. The explained variance is expressed by the Eigenvalues of a PC. The sum of all Eigenvalues corresponds to the variance of the dataset. The PCs are arranged according to the proportion of explained variance in descending order with the first principal component explaining the largest proportion of the variance and thus the strongest effect on ET.

For each PC, the projection of the original data onto the eigenvector yields a time series of coefficients, here referred to as scores. The scores thus represent the contribution of the PC to the variability of the data at each time step.

The magnitude and direction of the correlation between the original time series and a PC is referred to as a loading. The loading is calculated as the Pearson correlation of the z-normalised input data and the time series of the PC. It indicates how closely a time series (of ET values in a specific pixel) is associated with a pattern. This results in a compressed image of the spatial and temporal effects of key processes on ET.

Kröcher et al. (2025) conducted a PCA for Landsat ET data for the Lusatia region over a 30-year period from 1993 to 2022. These results were used in this study for the Landsat dataset. Two additional PCAs were performed analogously for the MODIS dataset in the 20-year period from 2001 to 2022 and the CERv2 dataset in the 30-year period from 1993 to 2022. We also compared the results with the spatial distribution of land use and with soil moisture time series.

## Results

### Spatio-temporal comparison of satellite- and model-based ET data

**Fig. 2:**
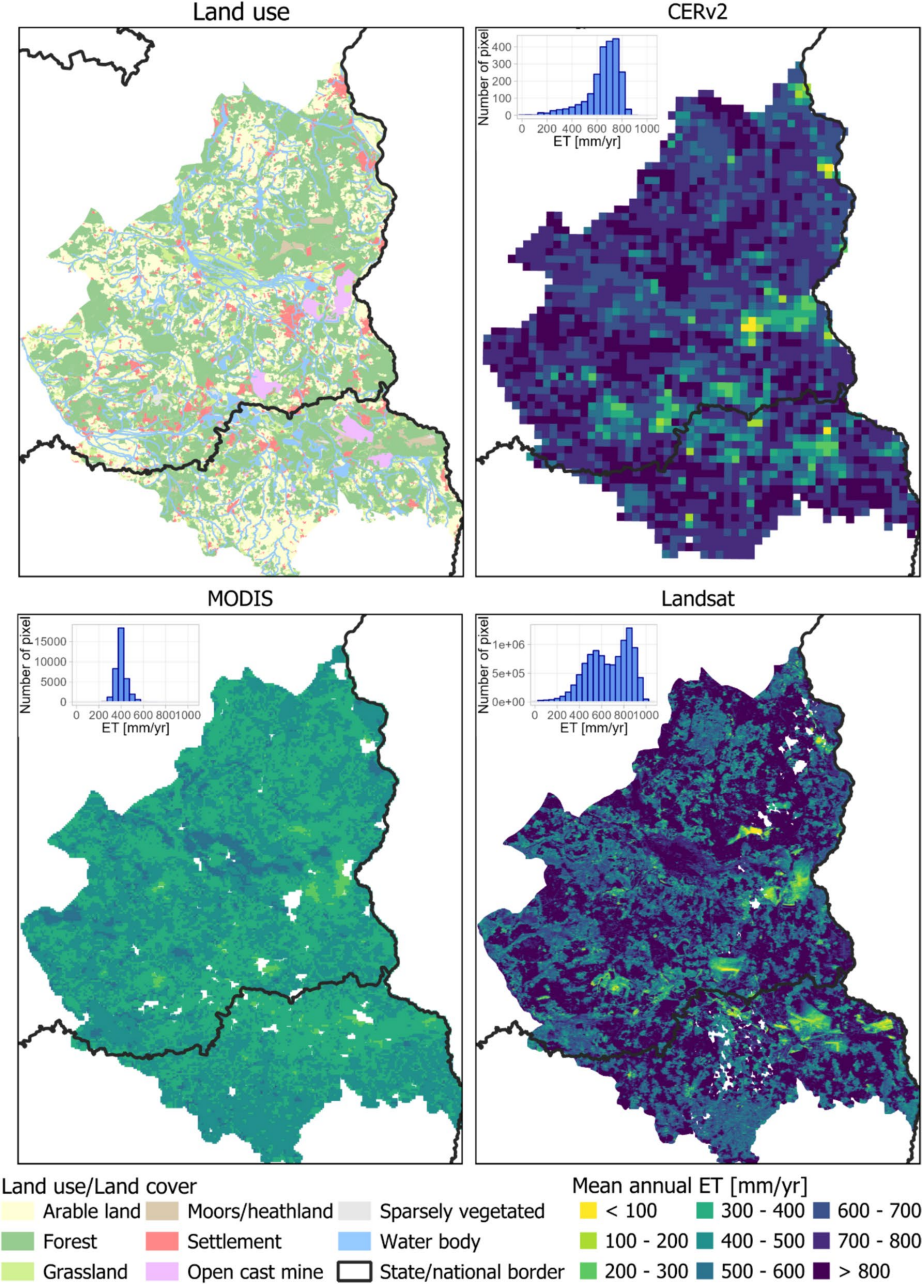
Land use and spatial distribution of mean annual ET of CERv2, MODIS and Landsat datasets from 2001 to 2022 over the study region

The spatial patterns of the mean annual ET were very consistent between CERv2 and Landsat. Both datasets estimate annual ET exceeding 800 mm yr^−1^, particularly in grassland and forested areas (Fig. 2). In contrast, the spatial distribution of annual ET in the MODIS dataset differs notably, with values ranging mainly between 200 and 600 mm yr^−1^. While MODIS also captures the highest ET rates in grassland areas with shallow groundwater levels, it yields much lower rates in forested areas. The Landsat dataset, conversely, shows a clear gradient between forested sites with values above 700 mm yr^−1^ and arable sites with values below 700 mm yr^−1^.

We performed a scale-sensitivity test by aggregating the 30-year averaged Landsat and MODIS product and comparing these aggregated grids to the original 30-year averaged Landsat and MODIS grids to quantify the scale effects. Table 3 summarises the scale effect expressed as the mean difference and standard deviation of annual ET between aggregated and native-resolution products. The uncertainty due to spatial resolution averaged between 0.0 ± 6.9 mm yr^−1^ and − 0.1 ± 24.6 mm yr^−1^ for the aggregated data (Table 3). When stratified by land use classes, the scale effects reached deviations of up to 24.4 mm yr^−1^, with standard deviations ranging from 6.0 to 47.2 mm yr^−1^. Mean values close to zero indicate that spatial aggregation alone introduces little systematic bias at the regional scale. However, the relatively large standard deviations demonstrate that aggregation can substantially affect local ET estimates, particularly within specific land-use classes. The highest uncertainties occurred in land-use classes with small spatial extent, particularly water bodies, sparsely vegetated areas, open cast mining sites and settlements. This reflects the increased mixing of contrasting surface conditions within larger pixels. By contrast, MODIS showed the smallest aggregation effects (up to − 2.0 ± 7.3 mm yr^−1^). This comparatively low sensitivity to aggregation can partly be explained by the smaller change in spatial resolution (aggregation factor of four from 500 m to 2 km), which reduces the degree of spatial averaging and land-use mixing relative to the aggregation of Landsat from 30 m to coarser grids.

**Table 3:** Mean and standard deviation (in brackets) of scale effects (aggregated Landsat 30 m/MODIS 500 m minus original 30 m/500 m grid) for different land-use classes at 500 m and 2 km grid resolutions

Land use	Scale effect [mm yr^−1^]		
	Aggregated Landsat 500 m to Landsat 30 m	Aggregated Landsat 2 km to Landsat 30 m	Aggregated MODIS 2 km to MODIS 500 m
Arable land	− 8.7 (18.7)	− 11.2 (19.6)	0.0 (6.6)
Grassland	− 2.8 (20.9)	− 3.7 (21.3)	− 0.1 (7.4)
Forest	7.1 (17.4)	9.8 (21.5)	0.1 (7.2)
Sparsely vegetated/moors/heathland	− 16.8 (25.9)	− 24.4 (47.2)	0.3 (7.3)
Settlement	− 7.6 (21.6)	− 16.0 (26.0)	− 0.3 (7.6)
Open cast mine	− 5.5 (19.6)	− 16.1 (26.6)	0.6 (6.0)
Water body	14.9 (19.4)	23.3 (20.0)	− 2.0 (7.3)
Overall area	0.0 (20.3)	− 0.1 (24.6)	0.0 (6.9)

### Comparison of satellite- and model-based with in situ ET data

#### Site-specific comparison of satellite, model and in situ ET

**Fig. 3:**
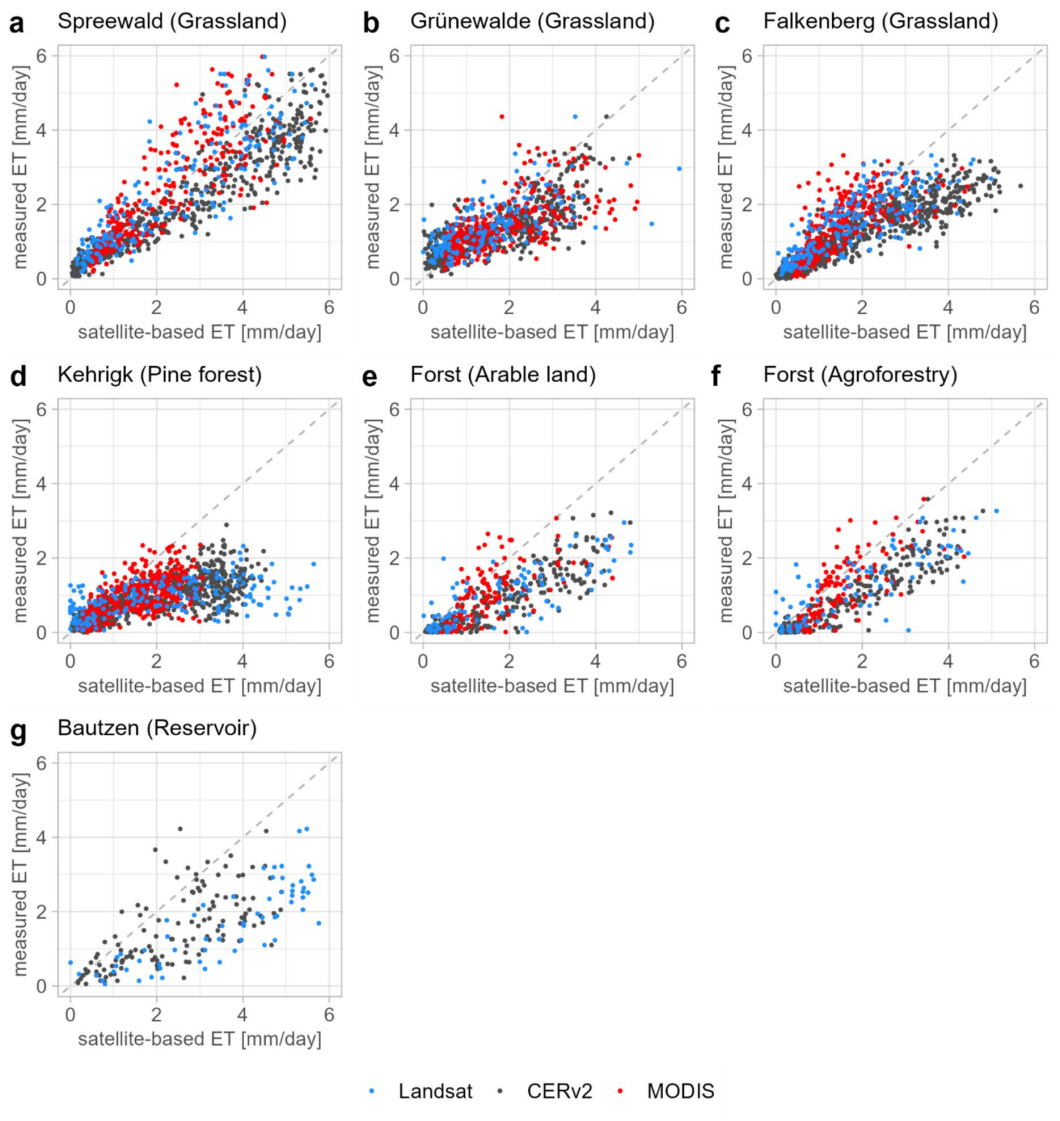
Comparison of weekly mean daily ET estimates from each satellite- and model-based dataset with reference measurements. The dashed 1:1 line indicates perfect agreement

The scatter plots and quality measures, which are based on comparison of weekly ET values, show varying degrees of agreement between satellite- and model-based and in situ measurements depending on land use and water availability at the sites. The lowest relative deviations are found at the Spreewald site (Table 4). The maximum ET values at the Spreewald site are consistently above 4 mm d^−1^ in the summer months in all years (Fig. 3a), while ET at the other sites remains below this threshold, with only few exceptions. These high ET rates are well represented by the satellite data, albeit with increasing dispersion as the ET rate increases. This location is characterised by a largely unlimited water supply of the vegetation due to a shallow ground water table. Thus, the ET process is only limited by available energy in terms of R_n_—G and the value of ET is very close to that of potential ET. This is properly reproduced by the satellite- and model-based datasets. In contrast, comparisons at the other sites, where the availability of water is a limiting parameter for the ET process, show overestimates by satellite data. At the two grassland sites Grünewalde and Falkenberg, our results exhibit low relative deviations between 5 and 54% (Table 4). However, ET measured in situ rarely exceeded 3 mm d^−1^, while Landsat and CERv2 systematically overestimate the daily totals of ET and frequently state ET rates of more than 5 mm d^−1^ (Fig. 3b–c).

**Table 4:** Statistical evaluation metrics for the comparison of each satellite- and model-based dataset against the in situ datasets

Measurement site (land use)	Dataset	Land use share in pixel [%]	R^2^	RMSD [mm d^−1^]	MAD [mm d^−1^]	BIAS [mm d^−1^]	Median relative deviation [%]
Spreewald (Grassland)	CERv2	39	0.86	0.92	0.66	0.51	21
	MODIS	90	0.69	0.86	0.61	− 0.23	10
	Landsat	100	0.64	1.02	0.73	− 0.12	8
Grünewalde (Grassland)	CERv2	18	0.63	0.80	0.61	0.35	19
	MODIS	44	0.41	0.90	0.65	0.41	21
	Landsat	100	0.45	0.79	0.59	0.11	5
Falkenberg (Grassland)	CERv2	6	0.86	0.99	0.69	0.66	54
	MODIS	27	0.55	0.58	0.41	0.09	11
	Landsat	100	0.66	0.72	0.52	0.27	13
Kehrigk (Pine Forest)	CERv2	96	0.63	1.3	0.94	0.91	80
	MODIS	100	0.40	0.8	0.62	0.54	54
	Landsat	100	0.24	1.86	1.39	1.18	96
Forst (Agroforestry)	CERv2	18	0.84	1.01	0.83	0.82	97
	MODIS	87	0.56	0.68	0.52	0.34	35
	Landsat	100	0.67	1.07	0.81	0.64	68
Forst (Arable land)	CERv2	76	0.79	1.13	0.91	0.9	116
	MODIS	77	0.46	0.82	0.64	0.51	41
	Landsat	100	0.63	1.29	1.00	0.89	78
Bautzen (Reservoir)	CERv2	57	0.41	1.36	1.08	0.9	75
	MODIS	85	-	-	-	-	-
	Landsat	100	0.60	2.12	1.90	1.83	118
Overall performance (pooled across sites)	CERv2	-	0.72	1.06	0.77	0.68	49
	MODIS	-	0.53	0.78	0.57	0.26	21
	Landsat	-	0.59	1.26	0.89	0.52	26

Similar overestimations with increasing ET rates, appear at the sites in Kehrigk, Forst and Bautzen (Fig. 3d–g). In particular at the Kehrigk and Bautzen sites the overestimation is considerably higher, even at lower ET rates. While in situ measurements in Kehrigk and Bautzen rarely exceed 2 mm d^−1^ and 4 mm d^−1^, respectively, the satellite datasets overestimate ET by 1 to 3 mm d^−1^ (Fig. 3d, g). In the MODIS dataset, ET was not estimated for water bodies, so no comparison for the Bautzen site is possible here.

**Fig. 4:**
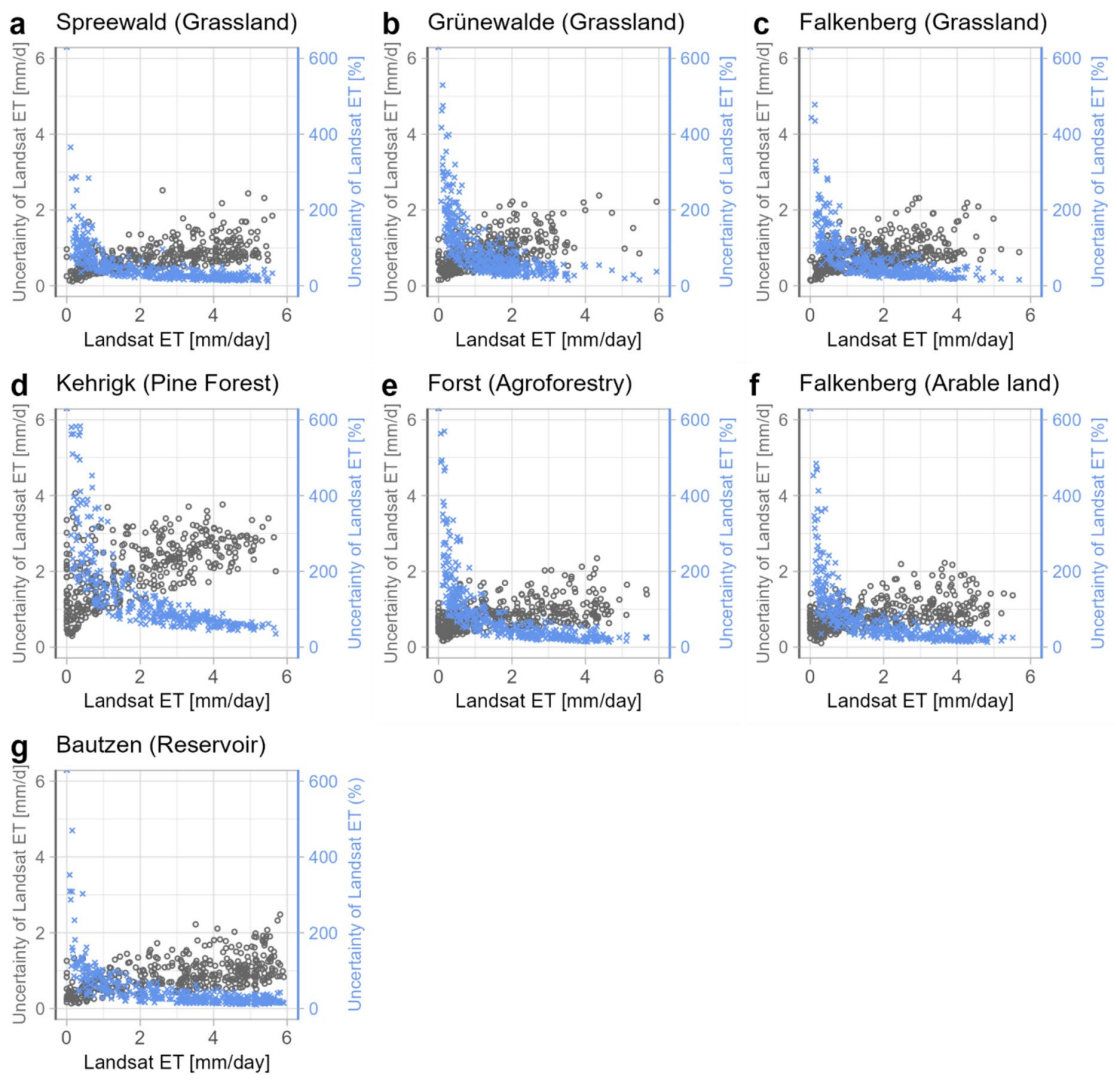
Uncertainties of Landsat ET in relation to Landsat ET values. Grey circles represent absolute uncertainty (mm d^−1^; left y-axis), and blue crosses represent percentage uncertainty (right y-axis)

Figure 4 shows the uncertainty as a function of the in situ measurements. Uncertainty assessments are provided alongside the ET values in the Landsat Collection 2 Provisional Actual Evapotranspiration Science Product. For small ET rates, the relative uncertainty exceeds 100% at all reference sites. While the relative uncertainty decreases with increasing ET rate and is below 100% from about 1 mm d^−1^ at most sites, the uncertainty remains high at the Kehrigk site (Fig. 4d), which is also consistent with the larger absolute deviations of ET from the in situ measurements.

#### Time-dependent comparison in ET

**Fig. 5:**
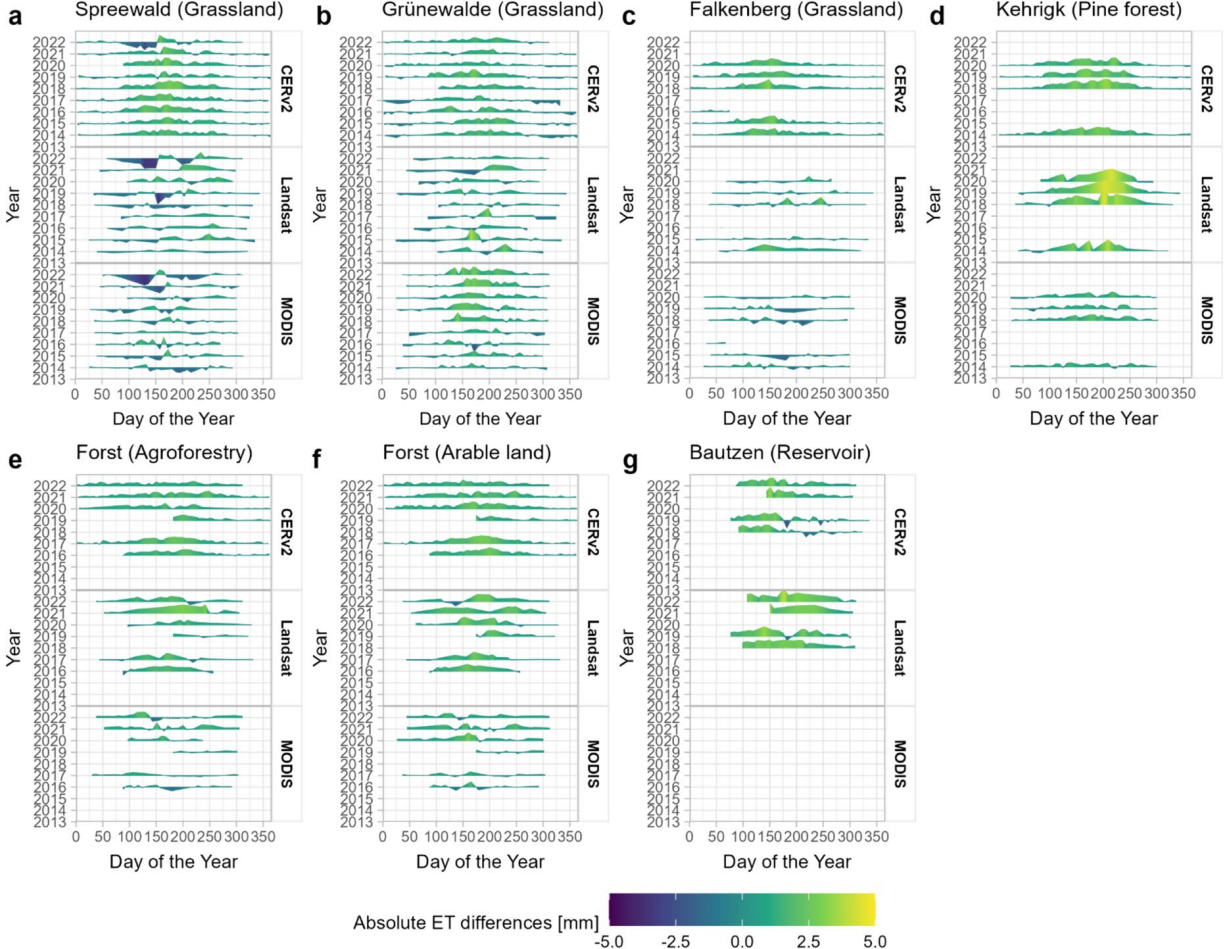
Temporal dynamics of absolute ET differences between satellite- and model-based estimates and in-situ measurements. Weekly aggregated differences are shown across multiple years (2013–2022) for each data source, with colour intensity indicating the magnitude of differences

The comparison of time series of reference measurements and satellite- and model-based datasets provides an overview of the extent to which systematic, seasonally dependent overestimations occur (Fig. 5). E.g., at the pine forest site in Kehrigk all three satellite datasets show large deviations between the 100th and 250th day of the year (Fig. 5d). At the grassland sites Spreewald, Falkenberg and Grünewalde, the CERv2 dataset shows largest differences between the 100th and 200th day (Fig. 5a–c). The MODIS and Landsat datasets, however, exhibit only sporadic, less systematic differences over the year. Notably, at the Spreewald site ET increased in May 2022 due to extensive growth of deep-rooting red clover in the lysimeter (Khaledi et al., 2024). However, as this small-scale vegetation change only appeared at the lysimeter and was not detected by the satellites, all satellite datasets underestimated ET in early summer 2022 (Fig. 5a).

The comparison of absolute deviations between the agroforestry site and the monocrop arable field site in Forst reveals that Landsat slightly overestimates ET at the monocrop site during late summer in dry years (2019, 2020, 2022) (Fig. 5e). This overestimation is less pronounced at the agroforestry site, likely due to the extended vegetation period of the trees beyond the crop harvest (Petzold et al., 2009). The strong overestimation at the agroforestry site in 2021 by Landsat (Fig. 5f) coincides with tree pruning in early spring, which resulted in reduced ET from the trees which was not captured by the Landsat dataset. For MODIS and CERv2, deviations between the two sites are less pronounced, though the reliability of both datasets may be constrained by their spatial resolutions of 500 m and 2 km, respectively, given the heterogeneous nature of this site.

### Principal component analysis

**Fig. 6:**
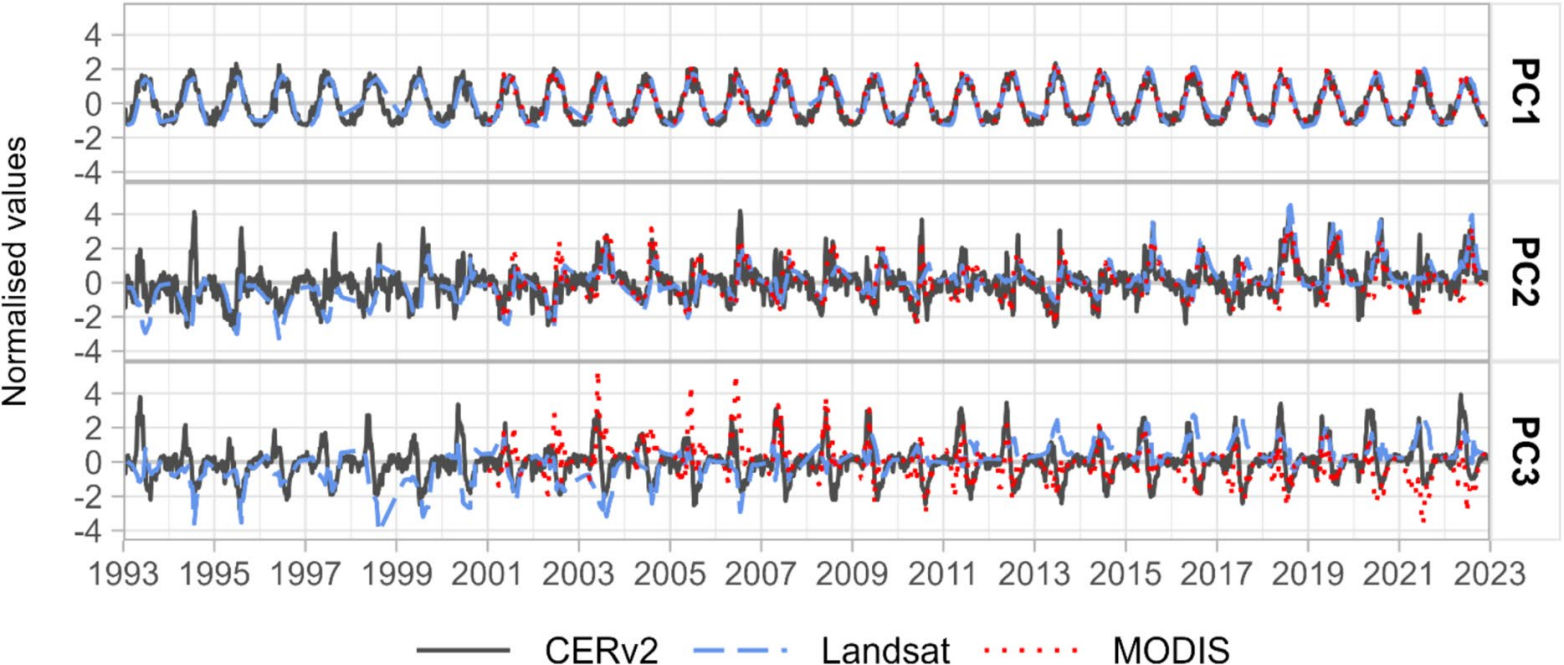
Time series of the scores of the first three principal components (PC1 at the top, PC2 at the middle, PC3 at the bottom) of the ET time series of CERv2 (grey, solid line), Landsat (blue, dashed line) and MODIS (red, point line) datasets

The PCA of satellite- and model-based ET time series resulted in the first three PCs explaining approximately 98% for CERv2, 86% for MODIS and 87% for Landsat of the total variance (Fig. 6). Each of these components exhibits distinct patterns as described by Kröcher et al. (2025).

#### First principal component

The first principal component (PC1) accounts for 97% (CERv2), 83% (MODIS) and 84% (Landsat) of the explained variance and captures the overall seasonal pattern of ET, characterised by high values in the summer months and low values in the winter months (Fig. 6). The PC1 time series of the three datasets show high positive correlation (MODIS and Landsat: *r* = 0.92; CERv2 and MODIS: *r* = 0.96; CERv2 and Landsat: *r* = 0.9).

**Fig. 7:**
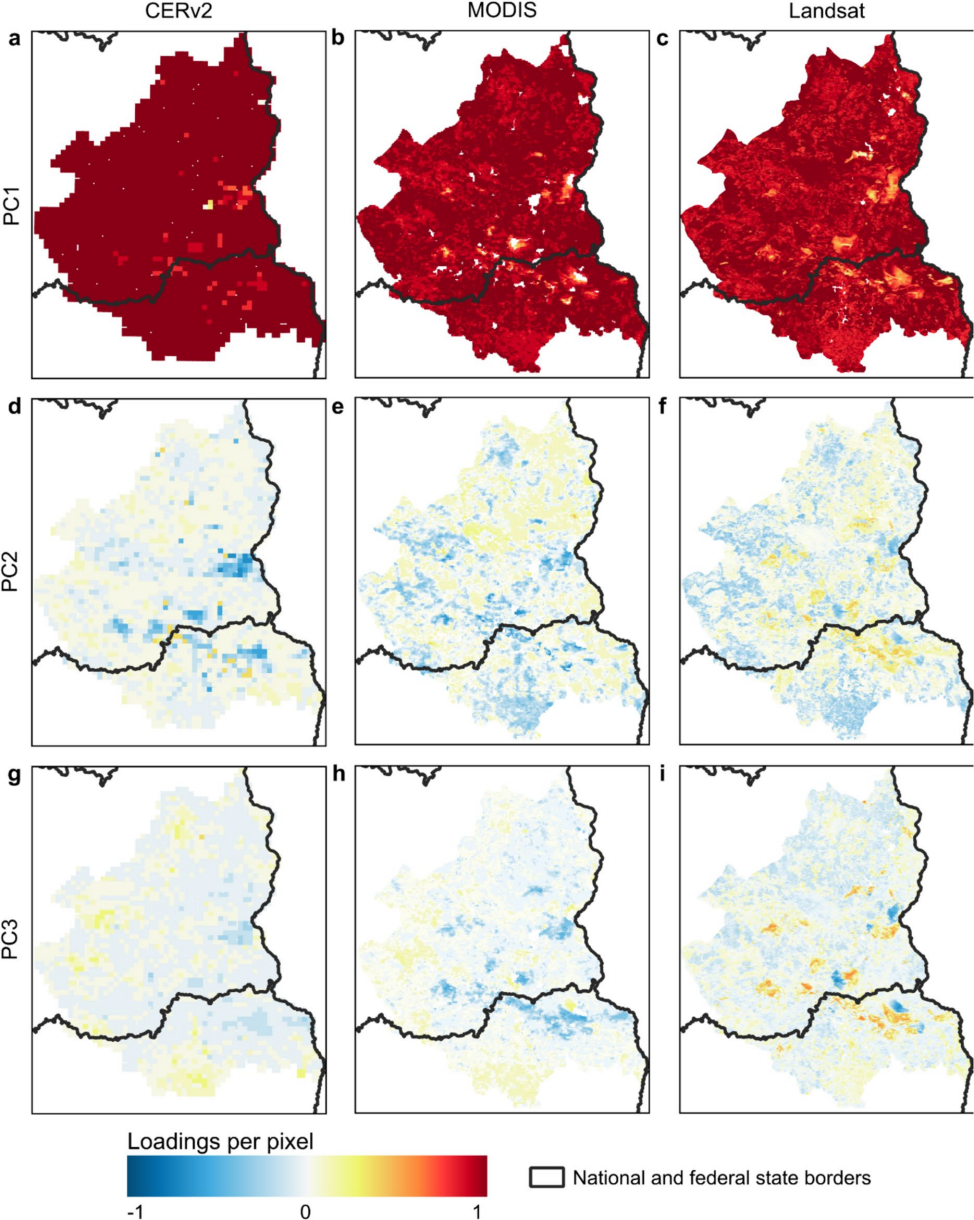
Spatial distribution of loadings on the three principal components (PC1, PC2 and PC3) of CERv2 (**a**, **d**, **g**), MODIS (**b**, **e**, **h**) and Landsat (**c**, **f**, **i**) datasets. The colour bar indicates the magnitude of the loadings per time series for each pixel

All datasets show high PC1 loadings that are relatively evenly distributed spatially (Fig. 7). Lower loadings occur in sparsely vegetated areas and opencast mines and therefore differ from the other areas in that the seasonal dynamics captures by the PC1 time series were less pronounced (Fig. 7).

#### Second principal component

**Fig. 8:**
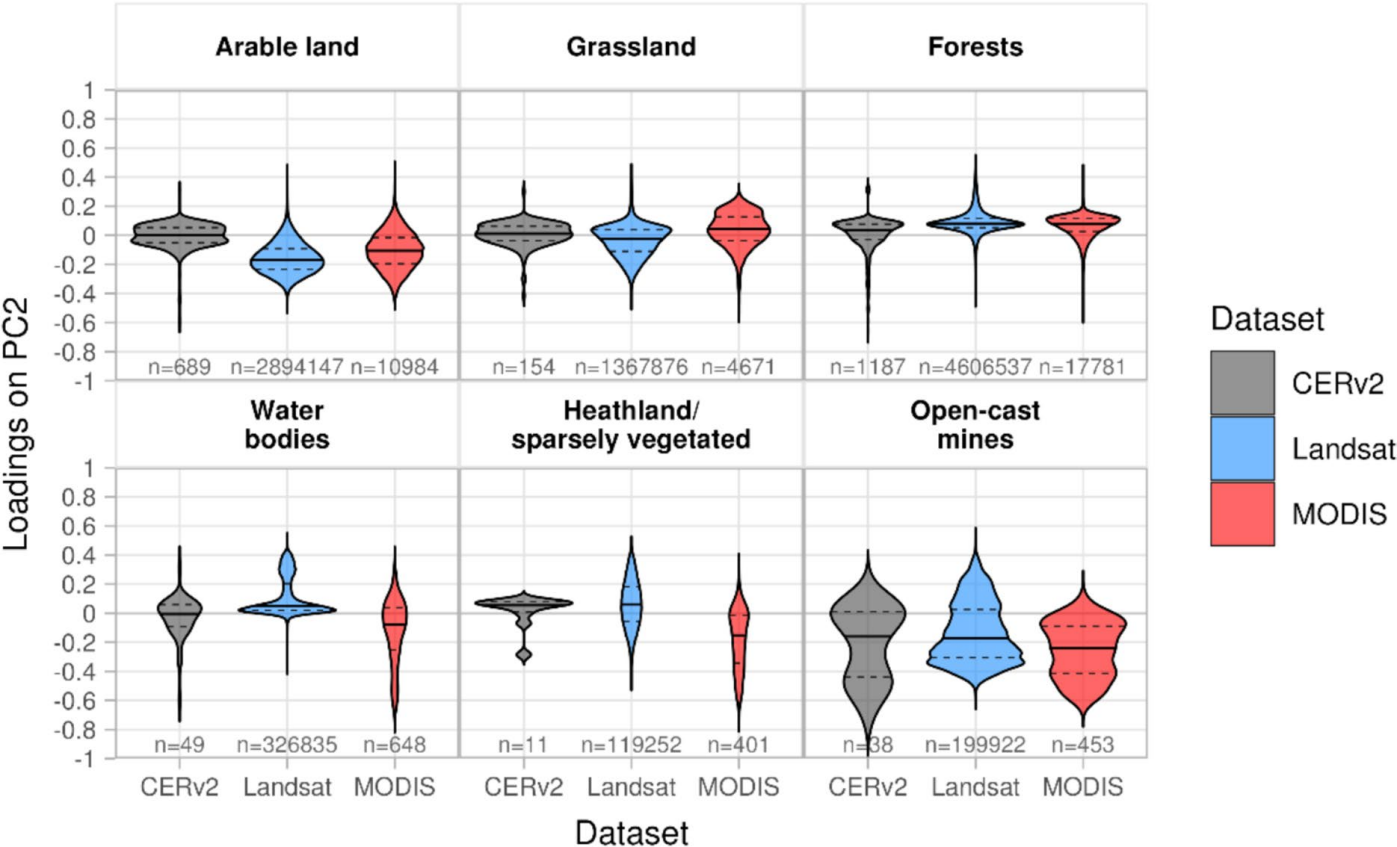
Distribution of loadings on PC2 for CERv2, MODIS, and Landsat-based datasets, grouped by the most frequent land use classes. Each violin plot shows the distribution of pixel-wise loadings; the solid horizontal line indicates the mean, and the dashed lines represent the 25th and 75th percentiles; ‘n’ denotes the number of pixels included in each category

The second principal component (PC2), which explains around 1% (CERv2), 2% (MODIS) and 2% (Landsat) of the variance, reflects a high spatial correlation with land use. Forest and water land uses stand out with loadings above zero, while arable land and opencast mines stand out with loadings below zero (Figs. 7f, 8).

The PC2 time series show an annual decline in spring with negative peaks in May and June, followed by a sharp increase and positive peaks in July and August (Fig. 6). During dry years, such as 2018–2020, the positive peaks are pronounced, while the negative peaks are less pronounced. Negative PC2 loadings, typical for arable land and opencast mines, therefore, indicate ET patterns with high ET in early summer and reduced ET in late summer, whereas positive loadings, typical for forests, grassland and water bodies, describe delayed ET peaks and higher ET values in fall compared to the mean pattern described by PC1.

The combined time series of PC1 and PC2 of all datasets shows moderate to high linear correlation with soil moisture time series at all sites in depths between 0.15 and 0.45 m (0.5 ≤ *r* ≤ 0.7), implying that PC2 captures the impact of soil water availability on ET patterns in different land use classes. Correlations between PC2 time series of all three datasets exceed *r* = 0.58.

#### Third principal component

**Fig. 9:**
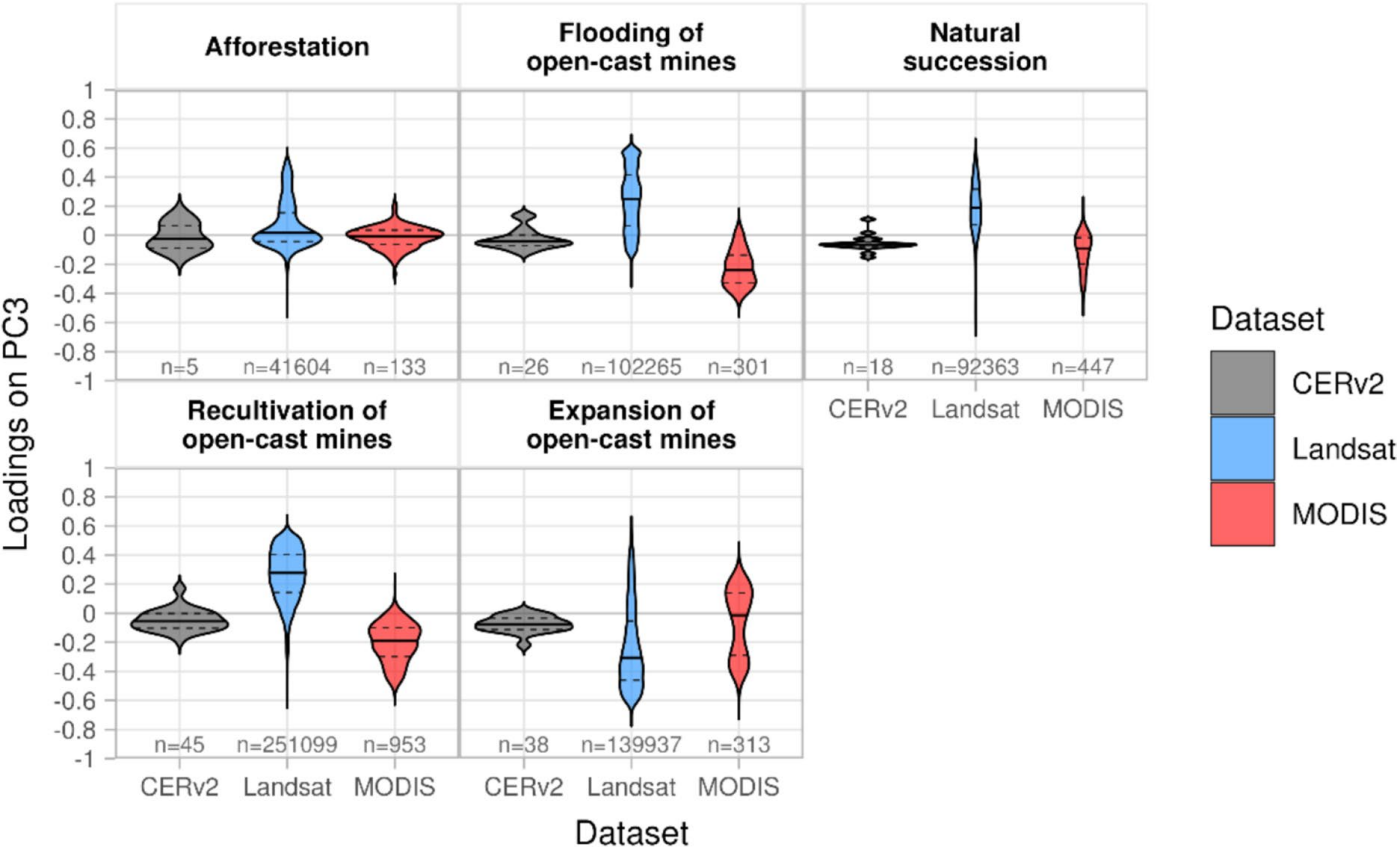
Distribution of loadings on PC3 of CERv2, MODIS and Landsat datasets, grouped by the most frequent land use change categories. Each violin plot shows the distribution of pixel-wise loadings; the solid horizontal line indicates the mean, and the dashed line represent the 25th and 75th percentiles; ‘n’ denotes the number of pixels included in each category

The time series of the third principal component (PC3) shows an increasing trend in ET peaks in summer for Landsat and a decreasing trend for MODIS, with less pronounced peaks during dry years (2018–2020, 2022; Fig. 6). For the CERv2 dataset, the long-term trend and the spatial distribution of high and low loadings are less distinct (Figs. 6, 7g, 9). Figure 9 shows the distribution of pixelwise PC3 loadings for the three datasets, grouped by the most frequent land use change categories. However, the distributions are affected by the number of pixels per category (‘n’), which varies strongly with spatial resolution and is relatively small for CERv2.

The spatial distribution of PC3 loadings for Landsat and MODIS reveal distinct patterns of high negative (MODIS) and positive (Landsat) loadings in areas undergoing natural succession, afforestation, land use restoration (e.g., former military training sites and closed open-cast mines) (Figs. 7h–i, 9). This suggests that vegetation regrowth leads to increasing ET trends over time here. In contrast, decreasing ET trends with positive (MODIS) and negative (Landsat) loadings appear in active mining areas (Figs. 7h–i, 9). This suggests that PC3 reflects long-term changes in ET driven by land use changes.

## Discussion

### Accuracy of satellite- and model-based ET estimates across land use types

The comparison between in situ and satellite- and model-based ET measurements shows varying degrees of accuracy depending on land use and water availability with the smallest relative deviations occurring at the grassland sites. Kalma et al. (2008) reviewed various remote sensing-based ET estimates and reported uncertainties ranging from 15–30% when compared to EC or Bowen ratio energy balance measurements, particularly in agricultural and grassland settings. The relative deviations at our grassland study sites—Spreewald, Grünewalde, and Falkenberg—fall within this uncertainty range (Table 4). Only the CERv2 data stand out with high deviations at the grassland site Falkenberg, which could be due to the high spatial heterogeneity of land use types, as grassland accounts for a small proportion in the CERv2 pixel (Table 4). The other sites show higher deviations but are still in the ranges as reported in García-Santos et al. (2022), who identified minor performance differences between thermal-based remote sensing models for different vegetation surfaces and spatial resolutions.

Beyond the effects of spatial resolution, systematic differences between ET products can be linked to their underlying algorithmic assumptions. Under energy-limited conditions, such as the water saturated grassland sites (Spreewald), all three products show relatively consistent ET estimates (Table 4). In contrast, under water-limited conditions, the differences between the products become more apparent, reflecting their varying sensitivity to soil moisture constraints and vegetation stress. These results underscore that the suitability of a product depends not only on spatial resolution but also on the prevailing hydrological regime.

CERv2 is based on a land surface modelling framework that relies on meteorological reanalyses and typically represents evapotranspiration as predominantly energy limited. As a result, CERv2 may overestimate ET under water-limited conditions where soil moisture constraints are not fully resolved at coarse spatial resolutions, especially in heterogeneous landscapes. MODIS can better account for seasonal water stress effects because it indirectly estimates water limitation through biome-specific parameterisations and atmospheric demand. This likely contributes to comparatively lower deviations across land use classes (Table 4). The Landsat SSEBop product is based on an energy balance approach that uses land surface temperature as an indirect proxy for evaporative cooling and moisture stress. While this enables the detection of fine-scale ET contrasts under water-limited conditions, water limitation is not explicitly modelled but inferred diagnostically from thermal signals. As a result, ET estimates can be sensitive to non-hydrological influences on surface temperature and to subpixel heterogeneity.

Sánchez et al. (2008) assessed Landsat ET estimates based on the Simplified Two-Source Energy Balance (STSEB) model and detected deviations of approximately 1 mm d^−1^ across different land uses. Our MAD values fall within these range, except for Kehrigk (Landsat) and Bautzen, where deviations are notably larger (Table 4). These systematic overestimations in the Kehrigk pine forest site may be caused by the pine trees adaption to the dry conditions and sandy soils by reducing transpiration under limited water availability — especially in late summer and early autumn (Irvine et al., 1998). These plant-physiological adaptations may not be fully accounted for in the parameterisations and algorithms utilised to create the datasets or pixel-based assumptions used in satellite-based ET models, especially in energy-balance approaches like SSEBop that do not distinguish between forest types (Mu et al., 2011; Senay et al., 2013). In contrast, MODIS, which uses biome-specific parameters predominantly estimates ET rates below 3 mm d^−1^ and exhibits smaller absolute deviations from the in situ reference measurements. This suggests that, despite its coarser spatial resolution, MODIS may better reflect the physiological constraints of transpiration in this ecosystem.

Further insights into the observed ET overestimations can be concluded from studies of Acharya and Sharma (2021) and Trebs et al. (2021) comparing different surface energy balance (SEB) models. They showed that in dry conditions, SEB models tend to overestimate ET while underestimating the sensible heat flux (Acharya & Sharma, 2021; Trebs et al., 2021). Although their studies focused on semi-arid regions, their findings are relevant in explaining our results. Trebs et al. (2021) specifically linked this overestimation of ET to an overestimation of aerodynamic resistance in arid, water-limited environments in Australia. Unlike the Penman–Monteith model used for MODIS ET estimates, which explicitly calculates aerodynamic and surface resistance (Cleugh et al., 2007; Mu et al., 2013; Zhang et al., 2016), the SSEBop model used for Landsat-based ET estimation does not include these parameters as direct inputs. This distinction may explain the comparatively better performance of MODIS and the overall lower mean annual ET in our study area (Fig. 4). Another important methodological issue arises from the scale mismatch between the spatial footprints of EC measurements and satellite pixels. While EC integrates fluxes over a dynamic footprint area which changes with wind direction, wind speed, and atmospheric stability (Oren et al., 2006), satellite observations typically capture different spatial extents. This makes direct comparisons challenging, particularly at shorter time scales. Nonetheless, the fact that Landsat with finer resolution, still overestimates ET, while MODIS showed overall less pronounced deviations to the in situ measurements, suggests that model approaches, rather than spatial resolution account for most of these differences.

Another site with high deviations between in situ and satellite- and model-based measurements is the reservoir at Bautzen. Water surfaces have a thermodynamically much more complex evaporation characteristics than land surfaces (Spank et al., 2025). The energy balance of water bodies strongly depends on heat storage in the uppermost layer (epilimnion), inducing substantial time delays compared to the atmospheric forcings (Spank et al., 2025) which is not accounted for in the Penman–Monteith approach. In addition, thickness of the epilimnion and stability of stratification varies over time.

Additionally, the smooth nature of the water surfaces leads to smaller eddies, increasing aerodynamic resistance and limiting turbulent exchange, which restricts the transport of water vapour from the water surface into the atmosphere. The strong horizontal energy exchange at the water body’s shore zone through advection also leads to considerable spatial heterogeneity in evaporation, which may be challenging to capture for satellite-based ET models at higher resolution (Raabe et al., 2026; Spank et al., 2025). Consequently, the satellite-based ET models, which are primarily designed for land surface processes, show poor performance for the Bautzen site. Nevertheless, we included this site in our analysis because lakes play an important role in the regional water balance and are of high relevance for water management in the Lusatia region. In addition, the reservoir site provides a useful test case for evaluating the limitations of satellite- and model-based ET estimates in environments dominated by non-terrestrial surface processes.

We focused on validation at the site level, as ground-based reference measurements are available only at specific locations. Although basin-scale validation using water balance approaches is conceptually possible, it was not conducted in this study for methodological reasons The anthropogenic influence in the study area has a direct impact on the water balance and thus ET. Besides land use changes, groundwater lowering and surface-water abstraction modify soil-moisture, resulting in highly variable and non-stationary ET patterns. These dynamics are difficult to quantify using conventional methods. Since spatially consistent groundwater or abstraction data are limited for the catchments in the study area, water balance calculations at the catchment scale would be subject to considerable uncertainty and are therefore unsuitable for robust validation of ET datasets. This data gap also highlights the importance of spatially comprehensive datasets, such as satellite-based ET estimates, especially in regions where conventional hydrological monitoring is limited by anthropogenic alterations. Future work could extend this analysis by incorporating additional hydrological datasets to enable catchment-level validation, which would provide valuable insights into the performance of satellite- and model-based ET datasets at regional scales.

### Uncertainty of reference and satellite- and model-based ET assessments

Ground-based reference measurements, such as those from EC flux towers and lysimeters, are crucial for validating satellite- and model-based ET estimates. While these measurements provide valuable insights, they are not free from uncertainty arising from different sources, which must be carefully considered.

For lysimeter measurements, systematic and random uncertainties arise from various sources, such as precipitation estimation, seepage water measurement, and unaccounted precipitation before or after rainfall events. Precipitation was determined based on the weight difference of the lysimeter to reduce inaccuracies resulting from the local precipitation gauge. This method also reduces uncertainties of precipitation gauges arising from wind effects, vegetation interferences and technical errors (Ciach, 2003; Schrader et al., 2013). However, the assumption of no ET before or after rainfall events, e.g., during summer heavy rain, can lead to an underestimation of ET. Hirschi et al. (2017) quantified this effect as an 8% underestimation for a lysimeter site in Switzerland. Allen et al. (2011) further summarised multiple systematic and random uncertainties in lysimeter-based ET measurements, suggesting a typical error range of 5–15%. Similarly, Seneviratne et al. (2012) and Hirschi et al. (2017) estimated an overall measurement uncertainty of 5–10%, with higher uncertainties observed during snow-covered periods. Despite these challenges, lysimeters remain one of the most precise methods for ET estimation when properly maintained and calibrated (Allen et al., 2011).

In the studies of Spank et al. (2013, 2016; 2020; 2023; 2025), the uncertainty and resulting tolerances of EC measurement data are carefully and comprehensively examined for both random and systematic uncertainties. Although the studies partly address other types of land cover and different scales of time, the general outcomes can be directly applied to this study. The EC measurement system likely underestimates actual ET (called ET_ref_ hereafter) by up to 20–40% (Finkelstein & Sims, 2001; Spank et al., 2025). This systematic underestimation arises primarily from flux contributions not accounted for by the EC methodology such as advective fluxes or large-scale circulations and secondarily from hardware and site-related heterogeneities and deficits in EC post processing (Spank et al., 2025). Based on the ET_ref_ and therewith included correction of the systematic underestimation of EC measurement data, the generalised measurement uncertainty can be quantified by ± 10% for daily values of ET_ref_ above 1 mm d^−1^ and by ± 0.1 mm d^−1^ for values less than 1 mm d^−1^. For monthly estimates, the uncertainty can be quantified as ± 15 mm based on a conservative approximation. However, lower limit needs to be accounted for 0 mm for monthly values less than 15 mm for reasons of plausibility.

In addition to the uncertainties inherent in in situ measurements, satellite- and model-based data also come with uncertainties caused by the spatial resolution and the different models used for ET estimation, which may not fully capture local-scale dynamics or microclimatic conditions measured on the ground. Several studies have compared satellite-based ET estimates with in situ measurements, highlighting variations in accuracy and precision depending on spatial resolution and algorithmic differences (Acharya & Sharma, 2021; Sharma et al., 2016; Trebs et al., 2021). Sharma et al. (2016) found that Landsat-based ET estimates exhibited lower deviations in heterogeneous landscapes compared to MODIS, with an overall difference of approximately 1 mm d^−1^. They attributed this primarily to MODIS’s coarser resolution, which aggregates land cover and smooths out local variations (Sharma et al., 2016). Although spatial resolution could influence the magnitude of discrepancies, isolating its impact from model-related differences is challenging.

The scale sensitivity analysis (Table 3) showed that spatial aggregation does not cause systematic bias in mean ET at the regional level, as the mean difference of the aggregated products across the entire study area is close to zero. However, aggregation increases the dispersion of ET estimates, which indicates a loss of spatial detail rather than a shift in average conditions. This effect becomes more pronounced with larger aggregation steps. Aggregation of Landsat from 30 m to 2 km results in standard deviations of up to 47 mm yr⁻^1^ for sparsely vegetated areas and more than 26 mm yr⁻^1^ for settlements and open-cast mining areas, while aggregation from 30 to 500 m results in lower standard deviations. These results show that scale effects are strongly influenced by the degree of spatial averaging and the heterogeneity of land cover within the aggregated pixels. In contrast, the aggregation of MODIS from 500 m to 2 km leads to consistently low deviations (standard deviation 6–7 mm yr⁻^1^ across all land use classes). This lower sensitivity may be directly related to the smaller aggregation factor and the already spatially smoothed nature of the MODIS product and does not necessarily indicate a generally higher robustness of the MODIS ET algorithm. The land use-specific results also show that scale effects are greatest for land cover types with strong subpixel ET contrasts, such as water bodies and sparsely vegetated areas, while more homogeneous land uses such as grassland and cropland exhibit lower aggregation-induced variability. This shows that spatial resolution primarily influences the representation of ET heterogeneity rather than the overall magnitude of ET.

In our study, the surroundings of all reference sites are characterised by spatially heterogeneous conditions. For instance, the EC measurements at Falkenberg are representative for a grassland patch of about 300 m × 150 m in size which is surrounded by arable land where different agricultural crops are grown each year (typically triticale, barley, rape seed or maize). These surrounding fields fall within the footprint of the corresponding MODIS and CERv2 pixels. Moreover, the grass at the Falkenberg field site is kept short by regular mowing such that measured ET there will certainly be smaller than for a reference grass surface (or for the surrounding farmland) due to significantly smaller LAI values. Similarly, the reference sites at Forst and Grünewalde include various small-scale variations of land cover and soil characteristics, contributing to spatial variations and therewith to the uncertainty of satellite- and model-based ET estimates.

The coarse spatial resolution of CERv2 inevitably leads to a lower representativeness of site-specific land-use conditions compared to MODIS and Landsat. Across all measurement sites, CERv2 pixels exhibited the lowest proportion of the dominant local land-use class, reflecting the heterogeneous land-use mosaic of the study region. This reduced representativeness likely contributes to the larger deviations observed at sites with strong surface heterogeneity, particularly agroforestry, arable land and reservoir sites. In such environments, ET is strongly controlled by fine-scale variations in vegetation structure, soil moisture availability and water management practices, which cannot be fully resolved at a 2 km grid spacing. Consequently, part of the observed ET deviations for CERv2 may reflect spatial aggregation effects rather than deficiencies of the underlying model formulation.

The temporal resolution of satellite- and model-based datasets also directly affects their ability to capture short-term variations in ET. As a reanalysis dataset, CERv2 integrates meteorological and satellite-derived inputs, enabling continuous daily estimates or even higher temporal resolution, while also minimising data gaps caused by cloud cover. The MODIS dataset, with an 8-day temporal resolution, is similarly well-suited to capture short-term fluctuations and seasonal trends. In contrast, Landsat has a revisit interval of 16 days, which may limit its ability to capture short-term ET changes, such as those caused by irrigation or heavy rainfall events. However, depending on the study period, combining observations from multiple Landsat generations (Landsat 4 to 9) can improve temporal resolution (Anderson et al., 2012). In addition, cloud cover significantly reduces the effective temporal resolution of optical satellite datasets, introducing gaps in time series and therefore affects the comparison with reference measurements.

The choice of weekly aggregation of time series inevitably smooths out short-term ET fluctuations, such as rapid responses to individual precipitation events or heat extremes. As a result, very short-term water stress signals may be dampened. However, the focus of this study is on evaluating longer-term ET behaviour, including seasonal patterns, interannual variations, and persistent drought effects, which are well preserved at weekly resolution. For the evaluation metrics (Table 4), weekly aggregation tends to stabilise deviations by reducing the influence of individual day outliers. For the PCA results, the weekly aggregated time series primarily reflect dominant temporal ET patterns rather than high-frequency fluctuations, which is consistent with the objectives of this study. Future work could explicitly evaluate fluctuations below the weekly level, where high-frequency ET dynamics are of interest.

Beyond spatial and temporal resolution, sensor performance differences introduce another source of uncertainty. Although Landsat and MODIS sensors undergo regular radiometric corrections, sensor performance and consistency in the time series as well as the impact of the satellite-based surface temperature product (Jaafar et al., 2022) may introduce additional variability to ET estimates. However, these effects are difficult to quantify due to the dominant influence of model-related differences. While Landsat provides a quantitative uncertainty estimate for ET, MODIS only includes quality flags, making it difficult to derive a direct uncertainty range. These factors highlight the importance of local calibration and validation to assess the suitability of satellite-based ET datasets for practical applications.

### Representation of spatio-temporal ET patterns by satellite- and model-based datasets

The PCA was conducted to comprehensively compare the three satellite- and model-based datasets and assess whether they consistently capture spatial and temporal ET patterns across the study region. By reducing various time series into time series that reflect their essential characteristics, PCA provides information about the spatial and temporal distribution of the dominant physical and anthropogenic processes and contributes to a comprehensive understanding of the system. The first three PCs of the respective PCAs conducted for each of the three datasets, CERv2, MODIS and Landsat, explained approx. 98%, 83% and 87% of the variance in the respective datasets, indicating that the dominant factors influencing ET patterns were represented. This high proportion of explained variance suggests that the key factors driving characteristic spatio-temporal ET patterns are captured similarly by all three datasets.

PC1 captured the regionally coherent seasonal ET cycle with peak values in spring and early summer and minimum values in winter, which are primarily determined by climatic influences. This component represents the dominant portion of the total explained variance in all three datasets.

Although PC2 and PC3 explain a much smaller proportion of the total variance compared to PC1, they are essential for identifying spatially limited hydrological or land-use changes. Even small-scale or short-term changes, such as groundwater depletion due to mining or land cover transitions, can strongly influence local ET dynamics. These effects are not effectively represented by PC1 but are captured in the lower-order components. Therefore, PC2 and PC3 provide valuable insights into hydrological variability at the landscape scale that goes beyond the dominant climate-driven signal. Deviations from the seasonal pattern occur, for example, in sparsely vegetated areas where PC1 loadings are less pronounced. This aligns with the results of Lei et al. (2016), who attributed reduced transpiration in such areas to limited vegetation cover resulting in weaker seasonal patterns. In the study area, sparsely vegetated areas are mainly attributed to anthropogenic interventions within the last few decades. The removal of vegetation leads to a decrease in transpiration, while recultivation or natural succession of formerly intensively used sites increases transpiration over a longer period. The effects of these processes on ET are represented by PC3.

PC2 represents the processes of soil moisture controlled shifts in annual ET peaks relative to climatically induced seasonality (PC1). In addition, PC2 shows long-term shifts on arable land, which are consistent with findings from Douinot et al. (2019) who reported that ET activity shifts earlier in the year over long term, with peaks in spring and early summer (until June) followed by a decline in July and August after harvest.

Landsat has the advantage of high spatial resolution and of covering a long period, enabling the detection of small-scale structures and long-term changes (Guerschman et al., 2022). Conversely, MODIS and CERv2 offer higher temporal resolution, making them valuable alternatives for applications requiring more frequent observations. However, CERv2 poorly captures the spatial patterns of PC3 observed in MODIS and Landsat, that is, the long-term increasing or decreasing evapotranspiration at specific sites. This limited representation is likely attributed to CERv2’s coarser spatial resolution, which constraints its ability to detect small-scale land use changes and the associated ET variations.

The overall high spatial consistency of ET patterns in our study aligns with findings from Stisen et al. (2021), who compared various remote sensing-based ET estimation methods. They found that the vegetation-driven MODIS product as the one applied in this study exhibited strong agreement with vegetation- and temperature-driven products, reinforcing the reliability of these datasets in capturing spatial ET dynamics. Stisen et al. (2021) also concluded that, despite differences in methodologies and uncertainties, common spatial ET patterns emerge across different approaches, further supporting the robustness of the observed trends and patterns in this study.

## Conclusion

This study aimed to assess the accuracy of ET estimates and the reproduction of the spatio-temporal variability of three publicly available satellite- and model-based ET datasets based on MODIS, Landsat, and CERv2. Regarding the first research question, comparisons of the satellite- and model-based with in situ measurements datasets showed that the quantitative correctness of ET depends strongly on water availability, while differences between land use types were of secondary importance. The highest differences were found for the pine forest, grassland and agricultural sites in the summer months from June to August, when actual ET was limited by soil moisture. All satellite- and model-based datasets tended to overestimate ET during these drought conditions. In contrast, at the Spreewald grassland site, where water limitation was absent, all satellite- and model-based datasets delivered the best agreement to in situ measurements.

Concerning the second research question whether satellite- and model-based data capture the key features of spatio-temporal ET variability, PCA revealed similar suitability of all datasets. The primary seasonal ET pattern and its spatio-temporal modifications due to land cover, water availability on terrestrial sites and long-term land use changes, were consistently captured across different spatial and temporal resolutions by all three datasets. However, the dataset differed in regard to less pronounced seasonal patterns due to coarse spatial resolution or model parameterization, which lead to systematic deviations. This can be particularly critical in applications such as agricultural water management or drought impact assessment, where seasonal peaks in ET determine irrigation requirements and water allocation. In our case, MODIS captured the overall seasonal dynamics more reliably, while Landsat provided higher spatial detail but tended to overestimate absolute ET.

Taken together, the results suggest that all three datasets show spatio-temporal consistency, making them useful for various applications depending on the required spatial or temporal resolution for the respective application: MODIS seems to offer the most balanced representation of ET at moderate resolution, making it suitable for regional-scale assessments. Landsat is best suited for detecting local land use effects and small-scale heterogeneity due to its high spatial resolution, though it tends to overestimate absolute ET. CERv2 captures short-term dynamics well, but at the expense of spatial detail.

Overall, this study contributes to the validation of satellite- and model-based ET datasets in Central Europe. These findings provide a solid basis for targeted applications in hydrological modelling, water resources management and land use planning and underscore the need to account for dataset-specific limitations, especially the systematic overestimation of ET under water-limited conditions when integrating satellite- and model-based ET data into applied environmental assessment.

## Data Availability

The Eddy covariance datasets at Forst site are available at: [10.5281/zenodo.4038399] (https:/doi.org/10.5281/zenodo.4038399) (Markwitz et al., 2020) and [10.25625/A2Z8T8] (https:/doi.org/10.25625/A2Z8T8) (Calleja-Rodelas et al., 2025). The lysimeter dataset at Spreewald site is available at: [10.4228/zalf-mf0v-jk94] (https:/doi.org/10.4228/zalf-mf0v-jk94) (Dietrich et al., 2024) All other Eddy Covariance and lysimeter datasets will be made available on request.
